# Dysentery; Its Cause and Treatment

**Published:** 1888-04

**Authors:** E. M. Bridgford

**Affiliations:** Mexico, Mo.


### DYSENTERY; ITS CAUSE AND TREATMENT.

*By E. M. Bridgford, M. D., Mexico, Mo.*

For Daniel's Texas Medical Journal.

IN a recent issue of "*Daniel's Texas Med. Journal*" I read a short article, written by Dr. S. H. Cowden, of Solgohacie, Ark., in which is recommended an injection of Mucil Amyli, Pulv Ipecac Morphia Acet in dysentery.

Now, while this form of treatment may be, (and doubtless is), palliative in its effects, it is far from being curative. When we are called to see a case of dysentery or any disease, we first ask ourselves : What is the character of the disease, and what is it produced by ? In regard to a definition of dysentery, Prof. W. C. Maclean, M. D., (Reynols System, Med. vol. i p 3 7 2), describes it as being "a specific febrile disease, characterized by nervous depression, by inflammation and sloughing of the glandular apparatus of the large intestines, by tormina and tenesmus, with scanty mucus and bloody stools of a peculiar odor, changing as the disease advances to serous, and giving off a gangreuous effluvia."

The causes of this disease are numerous. It is always found in malarial districts, in which there is always more or less vegetable decomposition. It can, according to the best authority, be propagated from one person to another by means of a contagious specific poison conveyed by the stools of a patient suffering from this disease through their being mixed with drinking water. For instance, if the discharges from a dysenteric patient be thrown in a water-closet, and by one means or another it is communicated to a cistern, thereby impregnating the water used by a family for drinking and cooking purposes, with the poison of the disease. Under such circumstances, as just described, we can at once see that a whole family may be stricken with dysentery by drinking water in which its poison exists. The treatment of acute dysentery is usually very simple. In the first place the hygienic condition of the patient should be improved; the drinking water should be pure; the room in which the patient stays should be well ventilated, and all decomposing vegetable and animal matter should be removed. The alimentry tract should be cleared, and by a saline purgative so as to remove any substance that would act as an irritant to the mucous membrane of the intestines.

This treatment to be followed by

℞ Bismuth Sub Nit., gr. xxx.
Morphia acet., grss.
M. ft. charts No vi.

If symptoms of malarial poisoning are very prominent, give tonic doses, (gr. 3–6) of quinine at short intervals. Large doses are not indicated, as dysentery is a disease characterized by nervous depression.

